# Impact of Institutionalisation of Births on Health Policies and Birth Registration in India

**DOI:** 10.5334/aogh.4474

**Published:** 2024-08-16

**Authors:** Sheetal Verma, Laxmi Kant Dwivedi, Ritul Kamal

**Affiliations:** 1 International Institute for Population Sciences (IIPS), Govandi Station Road, Deonar, Mumbai, Maharashtra, India and Directorate of Census Operations, Govt. of India, Lucknow, Uttar Pradesh, India; 2 International Institute for Population Sciences (IIPS), Govandi Station Road, Deonar, Mumbai, Maharashtra, India; 3 Directorate of Census Operations, Govt. of India, Lucknow, Uttar Pradesh, India

**Keywords:** Birth registration, health services, institutionalisation of births, Janani Suraksha Yojana, policy planning, CRVS, RBD Act, NFHS, India

## Abstract

*Background:* The Registration of Births and Deaths Act (RBD) of 1969 in India mandates continuous recording of vital events; however, after more than 50 years of its enactment, universality remains elusive. Birth registration, a fundamental right, is essential for demographic analysis and effective policy planning. Birth registration is closely linked to child development, access to healthcare, and other societal factors. Analysing its trends helps in designing targeted interventions and monitoring progress toward the Sustainable Development Goals (SDGs).

*Objectives:* This paper aims to analyse the changes in birth registration across Indian states. This paper also examines the impact of institutionalization of births on registration and underscores its significance in policymaking.

*Methods:* The study utilises data from the latest two rounds of National Family Health Survey (NFHS-4 & NFHS-5) to analyse birth registration trends in India. Multivariable logistic regression analysis was employed to examine the impact of place of delivery on birth registration.

*Findings:* The comparison of NFHS-4 and NFHS-5 data demonstrates varying birth registration rates across Indian states, with notable progress in some regions and persistent challenges in others. Multivariable logistic regression analysis highlights the significant influence of place of delivery on registration likelihood. The interaction between wealth and place of delivery suggests a mitigating effect, indicating that increasing institutional births has a positive impact on birth registration, with this effect being more pronounced at different levels of household wealth. It highlights that wealthier households were more likely to register births due to the higher rate of institutional deliveries.

*Conclusion:* India’s journey towards universal birth registration under the SDGs presents progress and challenges. NFHS data shows improvements in birth registration, but disparities still persist. Socio-economic status, place of delivery, and maternal education have strong influences on birth registration. Institutional deliveries significantly increase registration likelihood, facilitated by programs like Janani Suraksha Yojana. Integrating birth registration with health services enhances health data accuracy and service delivery. By prioritising targeted interventions, addressing social barriers, and leveraging existing programs, India can ensure that every child’s birth is registered, advancing towards a healthier, more equitable future.

## Introduction

The most important milestone in the history of vital statistics in independent India was the enactment of the Registration of Births and Deaths Act of 1969 (amended from 1 October 2023) [1]. The Registration of Births and Deaths (RBD) Act of 1969 in India mandates the compulsory, continuous, and permanent recording of vital events such as births and deaths. This act establishes a systematic framework for the registration of these events, thus ensuring the creation of an accurate and comprehensive record of the demographic landscape of India. The process involves the meticulous documentation of essential information related to each birth and death, including the date, time, and place of occurrence, as well as details about the individuals involved.

Birth registration trends serve as a critical tool for monitoring progress towards achieving universal birth registration, a key component of the Sustainable Development Goals (SDGs) [2]. Birth registration, apart from being a fundamental human right, also helps in ensuring that a child is officially recognised and that his/her rights are upheld. Birth registration data helps governments understand demographics and distribute resources effectively, which in turn leads to more targeted development programs in fields like education, nutrition, and immunization, etc. Simply put, birth registration empowers children, informs governments, and ultimately paves the way for better policy planning and brighter futures for children [3]. The *Lancet*, in two Series publications, have emphasized the importance of a robust Civil Registration and Vital Statistics (CVRS) system for children, adults, and governments. The publications termed the missing vital events from the CVRS systems a “scandal of invisibility” [4–6].

The permanence of the recording process ensures the creation of a lasting and reliable repository of vital statistics. This information is vital for demographic analysis, epidemiological studies, and policy evaluation. Moreover, it facilitates the issuance of legal documents such as birth certificates, which are essential for individuals to access various rights and services throughout their lives. Yet after more than 50 years of its enactment, birth registration in India has yet to achieve universality. Studying trends in birth registration across states over time in India serves a crucial purpose in understanding the dynamics of demographic data collection, aiding policymakers, researchers, and public health officials in making informed decisions. Variances in registration rates may be linked to factors such as education, wealth, and urbanization [7, 8]. Kumari concluded that birth registration is more of a privilege for children whose parents are educated and wealthy and who live in urban areas [7]. Mohanty identified maternal autonomy, institutional births, antenatal care-seeking behaviour, caste, religion, household wealth, and parental education as significant determinants of birth registration, which emphasises the multifaceted nature of these influencing factors [8]. This knowledge is pivotal for crafting targeted awareness campaigns and policies that address specific challenges in different states, fostering inclusivity and equitable access to birth registration services. Kumar used multilevel regression analysis to conclude that 25% of the variation in birth registration lies between states and that the remaining 75% lies within states [9]. Bhatiya concluded that India largely achieved an on-average improvement in birth registration at the national level and also for each state. The progress was slowest for children living in the poorest wealth quintiles [10].

Wealth index has been found to be positively correlated with birth registration and institutional deliveries, with the chances of an institutional delivery increasing monotonically among women from the poorest wealth quintile to the richest wealth quintile [11]. Socio-economic condition of the mothers has also been found to be associated with an increase in the use of maternal health services, with women belonging to higher socio-economic groups having a better chance of visiting a health facility [11, 12]. Women belonging to the middle, the richer, and the richest wealth quintiles were found to have, respectively, four times, five times, and seven times greater odds of delivering in institutions compared to women belonging to the poorest wealth quintile [11]. Women from lower wealth quintiles often endure multiple hardships in accessing proper maternity care, thereby significantly reducing the chances of institutional births [13]. Studies have found that women belonging to poor households, underprivileged socio-economic groups, and rural areas have significantly lower odds of having an institutional delivery, whereas women from the richest wealth quintile have the highest odds of institutional delivery [14].

Globally, around 166 million children under the age of 5 (approximately 25%) are not registered. Despite this, in most low and middle-income countries (LMICs), over 80% of all births take place in health facilities [15]. Registration of births allows for easier counting and tracking, which could ultimately be used to improve health outcomes. Almost one-fourth of countries worldwide lack quality data to monitor CRVS coverage adequately [15], which makes tracking SDGs more challenging. Registration of births within the facilities at the time of birth provides an opportunity for enhancing data reporting and data quality, which can be utilized directly by health systems for improved and targeted health care provision [15, 16]. Integrating birth registration and health policies has multifaceted benefits of guaranteeing the legal rights and entitlements of children and improving the health facilities available to children [15].

The objective of this paper has many folds. The first one is examining the change in the level of registration of births across different states of India between two survey points of NFHS-4 and NFHS-5—that is, 2015–2016 and 2019–2021, respectively. The second is studying the interplay between wealth status of households and places of delivery in explaining the variation in the registration of births. This paper will help develop the understanding of the dynamics between these characteristics, which is crucial for better policy formulation and for unravelling the disparities in the registrations of births.

## Methodology

The paper has relied on comparisons of birth registrations through the usage of National Family Health Survey (NFHS) data. NFHS follows a uniform survey methodology across the country and over time. The NFHS also serves as the primary data source, thus offering a comprehensive understanding of birth registration trends in India. NFHS surveys, conducted in multiple rounds, provide representative samples of households across all states and all districts in India. The study focuses on the data collected during two rounds: NFHS-4 (2015–2016) [17] and NFHS-5 (2019–2021) [18]. NFHS-5, conducted in two phases between 2019 and 2021, covered all 707 districts in 28 states and 8 Union Territories (UTs), while NFHS-4 data collection occurred from January 2015 to December 2016.

The sampling procedures and sample size selection for both NFHS rounds are detailed in the respective NFHS reports (NFHS-4 report [17] and NFHS-5 report [18]). The International Institute for Population Sciences (IIPS) conducted NFHS-4 in 2015–2016 and NFHS-5 in 2019–2021, providing vital health and family welfare data. The number of households interviewed was 601,509 in NFHS-4 (2015–2016) [17] and 636,699 in NFHS-5 (2019–2021) [18].

## Study Variables

The primary outcome variable in this analysis is birth registration, considered a fundamental means of safeguarding a child’s right to identity and other associated rights. The NFHS collects data on the number of de jure children under age five registered by the civil registrar. The survey includes a question regarding birth registration: “Does a child have a birth certificate or has the child’s birth ever been registered by the civil authority?” [NFHS-4 and NFHS-5]. Responses are recorded in four categories: “neither certificate nor registered,” “has certificate,” “registered,” and “don’t know.” For the purpose of this study, we have clubbed data related to “has certificate” and “registered” since the primary goal of the exercise is to record the event of birth when it occurs.

In addition to birth registration, several demographic and socio-economic variables are considered in the analysis to identify factors influencing birth registration levels. These variables include the sex of the head of the household (male or female), the religion of the head of the household (categorised as Hindu, Muslim, and Other), the Caste of the head of the household (categorised as Scheduled Castes, Scheduled Tribe, and Other), wealth index (as given in NFHS), health insurance (yes or no), the structure of the household (nuclear or non-nuclear), education level of mother (No Education/Primary/Secondary/Higher), and place of delivery (institutional or non-institutional). The wealth index, is a composite measure of a household’s cumulative living standard. The wealth index is calculated using easy-to-collect data on a household’s ownership of selected assets, such as televisions and bicycles; materials used for housing construction and types of water access and sanitation facilities. The wealth index is divided into quintiles: poorest, poorer, middle, richer and richest [19].

The last-mentioned variable, namely, institutionalisation of births (deliveries), has never been studied in detail as a possible strong influence on birth registration, especially in view of the fact that in the majority of states in India, government medical institutions have been designated as “Registrars” in themselves.

## Statistical Analysis

Descriptive statistics are employed to present the background characteristics of the study samples. Bivariate analysis is utilized to understand the levels of birth registration across selected variables. This analysis involves examining the association of demographic, socioeconomic, and health care variables with children’s birth registration. A multivariable logistic regression model was employed to examine the interplay between wealth status of household and place of delivery on registration of births. Multivariable logistic regression analysis enables us to analyse the likelihood of birth registration based on various predictor variables. Logistic regression analysis is used to estimate the effects of factors when the independent variables are categorical. The multivariable logistic regression analysis is used to estimate the adjusted odds of outcome after controlling for confounding factors. P-value of <0.05 was considered to be statistically significant. All statistical analyses are conducted using STATA 18 [20], and the findings on birth registration levels are presented using Datawrapper [21], facilitating a comprehensive visualisation of the data.

## Results

**Figure 1:**
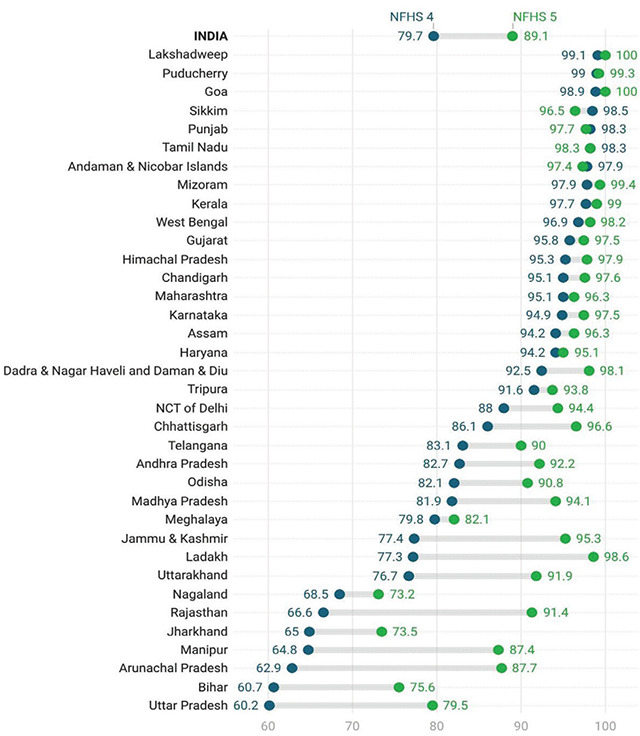
Birth registration (%) in NFHS-4 and NFHS-5 across States & UTs in India.

The National Family Health Survey (NFHS-5) provides information on births registered within one year, and it has been used to estimate the birth registration rate. The data from the National Family Health Surveys (NFHS) (Figure 1) reveals a mixed picture of birth registration in India, emphasising the need for targeted efforts in specific regions. In NFHS-4, Puducherry and Lakshadweep exhibited almost 100% birth registration, highlighting their efficient civil registration systems. In contrast, states like Rajasthan, Uttar Pradesh, Bihar, Arunachal Pradesh, Nagaland, Manipur, and Jharkhand reported birth registration rates below 75%, indicating significant gaps in the registration process. A majority of states and UTs, however, boasted birth registration rates exceeding 80%, emphasising a relatively widespread adherence to registration norms.

NFHS 5 data affirms that universal birth registration in India is an achievable goal in the near future. NFHS-5 data highlights significant progress in birth registration, with Goa, Lakshadweep, Kerala, Puducherry, and Mizoram achieving nearly 100% registration. Nevertheless, challenges persist in states like Nagaland, Jharkhand, Bihar, and Uttar Pradesh, where more than a quarter of births remain unregistered. Rajasthan, Arunachal Pradesh, Manipur, and Uttar Pradesh showcase noteworthy improvements, reflecting a positive trend in birth registration rates.

The focus on improving birth registration in the North Eastern States of Meghalaya, Nagaland, Arunachal Pradesh, and Manipur, as well as the expansive areas covered by Uttar Pradesh, Bihar, and Jharkhand, could significantly contribute to achieving universal birth registration. Notably, Uttar Pradesh and Ladakh have seen a remarkable increase of nearly 20%, and Bihar has seen an increase by 15% in birth registration rates between NFHS-4 and NFHS-5. However, states like Jharkhand, Nagaland, Meghalaya, and Tripura still face challenges and need to intensify their efforts towards achieving universal birth registration. Rajasthan and Arunachal Pradesh stand out, with a 25% jump in birth registration rates in less than a decade, showcasing remarkable improvement.

Table 1 shows the percentage of birth registration by selected characteristics of household and women.

**Table 1:** Comparative analysis of factors affecting birth registration (%) in NFHS-4 & NFHS-5.

INDICATORS	NFHS-5	NFHS-4	RELATIVE INCREASE (%) FROM NFHS-4
**Sex of Head of Household**			
Male	89.6	80.6	11.17
Female	87.3	77.3	12.94
**Religion of Head of Household**			
Hindu	89.1	80.1	11.24
Muslim	89.1	77.6	14.82
Others	92.7	89.5	3.58
**Caste of Head of Household**			
Scheduled Caste	87.9	79.2	10.98
Scheduled Tribe	88.1	76.4	15.31
Others	89.4	80.5	11.06
**Wealth Index**			
Poorest	81.1	64.6	25.54
Poorer	87.8	77.8	12.85
Middle	91.8	84.5	8.64
Richer	93.7	88.8	5.52
Richest	95.5	93.2	2.47
**Health Insurance**			
No	87.4	77.7	12.48
Yes	92.6	82.1	12.79
**Household Structure**			
Nuclear	86.8	77.2	12.44
Non-nuclear	90.7	82.1	10.48
**Education of Mother**			
No education	78.4	64.4	21.74
Primary	87	79.3	9.71
Secondary	92.6	88	5.23
Higher	94.5	91.7	3.05
**Place of Delivery**			
Non-institutional	68.8	57.7	19.24
Institutional	91.8	85.9	6.87

In both NFHS-4 and NFHS-5, male-headed households witnessed a slightly higher rate of birth registration than did female-headed households. This likely reflects the influence of patriarchal social norms, which continue to affect women’s access to civil registration authorities. It needs to be mentioned that the gap between the registration rates of the two categories has, per se, declined. NFHS- 4 showed a slightly lower birth registration percentage for Muslim households, which has evened out in NFHS-5, and religion seems to have lost significance as a differentiating factor on birth registration rates. This trend could be a result of growing coverage of civil registration institutions, as witnessed in Figure 1. The period between the two rounds of NFHS has also witnessed a decline in the influence of caste as a factor that could impact birth registration. Today, nearly all castes have a comparable birth registration rate as per the NFHS-5 results, a fact which once again points to the growing coverage of civil registration institutions and practices. Increasing levels of Wealth have been known to exhibit a positive correlation with birth registrations. However, two results are of note in the intervening period between NFHS-4 & NFHS-5. Not only has the gap in registration rates witnessed a decline between the two successive categories, but the gap between the poorest and the richest households in percentage points has also fallen sharply, from 28.6 percentage points in NFHS-4 to 14.4 percentage points in NFHS-5. This suggests that efforts may be working to bridge the access gap for birth registration across economic strata. Secondly, there is a remarkable increase of 25.0 percentage points in the birth registration rates for the poorest quintile between the two rounds of NFHS under study. It would thus be worthwhile to explore any regional or state-level variations in these trends.

**Figure 2:**
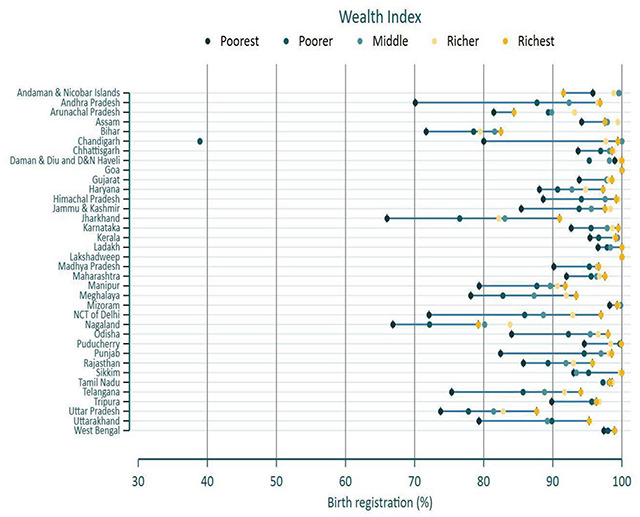
Equiplot presenting the birth registration (%) by Wealth index in NFHS-5.

Figure 2 completes the picture just given by reflecting upon the fact that the disparities in birth registration attributable to wealth inequalities have declined in all but the 10 states where the poorest households still have less than 80% of births being registered. Except for Jharkhand and Nagaland, no other state in the country has exhibited less than 70% of birth registration, even for the “poorest” category. This points to systemic inadequacies which can’t be attributed solely to factors regarding the household (demand) side.

It is also important to highlight the wide disparities across wealth quintiles in the states of Andhra Pradesh, Jharkhand, Chandigarh, Delhi, and Telangana. These five states have a variation of nearly 20 percentage points between the poorest and the richest households. It reflects the fact that although the systems for birth registration are well in place, there is an access issue for the poorest households, which could be physical as well as financial. In the states of Uttar Pradesh and Bihar, even the richest households have reported less than 90% registration, which not only points to the fact that these two states were way behind the national average for a long time but also, in a way, highlights some underlying issue (possibly out-migration, which led households to express their inability to comment on the registration status of the children born in the family). The five-percentage-point differential in birth registration rates in households with and without health insurance has also persisted in both rounds of the NFHS. Non-nuclear households have exhibited a better percentage of birth registration rates than nuclear households in both rounds, although the gap has narrowed over the years, once again points to increasing coverage of civil registration over the years. Education of mothers has also been known to have a positive correlation with birth registration rates previously. However, one needs to look at the fact that while there was a 27.3% differential in birth registration rates between the category of mothers who had no education and those who had attained higher education, this differential has narrowed to 16.1% over the years under study. Lastly, but astonishingly, NFHS-4 results show that the difference in registration rates of institutional and non-institutional births is a whopping 28.2%. Even in NFHS-5, the difference has only marginally narrowed to 23.0% despite the fact that all other variables previously mentioned have pointed towards a growing coverage of civil registration institutions. In view of the massive differential which persists in this category, a detailed analysis was carried out.

Finally, multivariable logistic regression was carried out to examine the association between selected variables and birth registration (Table 2). None of the variables identified in the study, except religion, was found to be statistically not significant. As surmised above, place of delivery has the strongest influence on birth registration. So much so that, per NFHS-5, an institutional birth had three times more odds to be registered than a non-institutional one.

**Table 2:** Logistic regression analysis of demographic and socio-economic factors associated with birth registration in NFHS-5.

PARAMETERS	ODDS RATIO	95% CONFIDENCE INTERVAL LOWER-UPPER
**Sex of Head of Household**		
Male	Reference	
Female	0.87 ***	0.84–0.91
**Religion of Head of Household**		
Hindu	Reference	
Muslim	1.03	0.98–1.07
Others	0.98	0.93–1.03
**Caste of Head of Household**		
Scheduled Caste	Reference	
Scheduled Tribe	1.25 ***	1.19–1.31
Others	0.95 *	0.92–0.99
**Wealth Index**		
Poorest	Reference	
Poorer	1.30 ***	1.22–1.38
Middle	1.58 ***	1.45–1.74
Richer	1.89 ***	1.66–2.15
Richest	2.00 ***	1.64–2.44
**Health Insurance**		
No	Reference	
Yes	1.49 ***	1.45–1.54
**Household Structure**		
Nuclear	Reference	
Non-Nuclear	1.04 **	1.01–1.07
**Place of delivery**		
Non-Institutional	Reference	
Institutional	2.93 ***	2.80–3.07
**Education of mother**		
No Education	Reference	
Primary	1.43 ***	1.36–1.49
Secondary	1.86 ***	1.79–1.92
Higher	1.74 ***	1.63–1.85
**Interaction between Wealth Index and Place of Delivery**		
poorer*institutional delivery	1.08	1.00–1.16
middle*institutional delivery	1.15 **	1.04–1.28
richer*institutional delivery	1.22 **	1.06–1.41
richest*institutional delivery	1.63 ***	1.32–2.01

* p < 0.05, ** p < 0.01, *** p < 0.001

It was pertinent to run a logistic regression with an interaction term to study the interaction effect of two variables, namely, wealth index and place of delivery, to arrive at a more nuanced understanding of how economic status of household influenced birth registration through choice of place of delivery. The interaction was found to be statistically significant. This translates to the fact that wealth and institutionalisation of deliveries have a mitigating impact on each other. The interaction between wealth and place of delivery suggests a mitigating effect, indicating that increasing institutional births has a positive impact on birth registration, with this effect being more pronounced at different levels of household wealth. By providing financial assistance for institutional deliveries, Janani Suraksha Yojna (JSY) [22] has helped bridge the affordability gap for economically disadvantaged women since 2005. The impact of this scheme across the country is visible. A notable consequence of institutionalized births is the potential enhancement of birth registration rates. Institutional deliveries occurring within health care facilities typically result in higher rates of registration because of the structured documentation procedures inherent in such environments. Health facilities thus serve as key initiators of the birth registration process, ensuring the comprehensive recording of vital information. Even if the civil registration authorities are situated outside the hospital premises, health facilities facilitate the reporting of this crucial data, thereby bolstering the registration process.

## Discussion

India’s journey towards universal birth registration, a crucial component of the SDGs, presents a complex picture of progress and challenges. While significant strides have been made in recent years, disparities across states still persist. This study, analysing data from two rounds of the National Family Health Surveys (NFHS-4 and NFHS-5), sheds light on these variations and highlights the interplay between institutionalized deliveries and the economic status of household on the registration of births in India. Overall, there has been a notable improvement in birth registration rates across India. States like Uttar Pradesh, Bihar, Arunachal Pradesh, Manipur, and Jharkhand, though still lagging behind, have witnessed positive increases.

Studies have highlighted the challenges faced by children without birth registration, emphasizing the importance of birth certificates for accessing public schemes and facilities. Similarly, research in low- and middle-income countries (LMICs) has shown a negative association between lack of birth registration and early child growth and development outcomes. Integrating birth registration with early child health, nutrition, and education programs is crucial for ensuring that every child is legally recognized and has an equal opportunity to reach their full developmental potential [23]. The study also highlighted the role of sex of head of household in birth registration. Birth registration in female-headed households has increased from 77.3% in NFHS-4 to 87.3% in NFHS-5. The results of our study are consistent with the results of the study of national surveys from 93 low- and middle-income countries, which found that female-headed households (with no male member) had lower adherence to birth registration as compared to male-headed households [24].

The study presented compelling evidence that wealth status of household through institutional births exert the strongest influences on birth registration in India. Results clearly indicate that the interaction between wealth status of households and births delivered in an institution played positive significant roles in improving the number of registrations of births. The institutional delivery rate in India has seen a substantial increase, nearly doubling from 38.7% in NFHS-3 to 78.9% as per NFHS-4. Initiatives like the Janani Suraksha Yojana (JSY) [22, 25], which incentivise institutional deliveries, have played a pivotal role in this improvement [25]. Additionally, schemes like the Janani Shishu Suraksha Karyakram (JSSK), which providing free transport, food, drugs, and consumables to pregnant women and infants, have contributed to the rise in institutional deliveries by reducing out-of-pocket expenditures [26].

Generally, wealthier households have better access to health care facilities due to financial resources. They are more likely to opt for institutional deliveries, where registration processes are integrated into the health care systems. It is also observed that wealthier households also often have higher levels of education and greater awareness about the importance of birth registration. They are more likely to understand the benefits of registering births and are proactive in complying with the registration requirements.

Overall, the interplay between institutionalized deliveries and household wealth status on higher registration rates underscores the complex relationship between socio-economic factors and health care utilisation. Addressing disparities in access to health care and improving awareness about birth registration among disadvantaged communities are essential steps towards achieving universal registration coverage.

The significance of birth registration extends beyond individual well-being. Birth certificates obtained through institutional birth centers serve as gateways to improved health, education, and social protection for children. They facilitate access to vital health care services such as immunizations and school enrolment and also establish legal identity, thus enabling individuals to claim for social protection schemes and entitlements. Moreover, birth registration empowers policymakers by providing accurate demographic and health data, enabling evidence-based initiatives for child welfare, and contributing to a healthier, more equitable society.

A fully functional Civil Registration and Vital Statistics (CRVS) system, ensuring complete birth registration, offers multifaceted benefits. It addresses the “denominator problem,” improves the accuracy of health statistics, and provides a demographic base for planning health care service delivery. Integrating birth registration within public health and social welfare schemes has proven to be a cost-effective approach in enhancing large populations’ access to services. A computerized CRVS system integrated with various welfare schemes thus serves as the backbone of policy planning, providing access to real-time data, improved evaluations, and enhanced monitoring of programs [27].

## Conclusion

India is home to around 18% of the global population; however, India has a disproportionately high percentage of morbidity and mortality among children and pregnant women, both of which can be prevented by interventions such as institutionalisation of deliveries, access to ante-natal and post-natal care for women, immunization of children, etc. [28]. India’s full immunization coverage stands at 76.1%, according to NFHS-5 (2019–21) data, meaning that one out of every four children is missing out on essential vaccines. Gaps in immunization coverage likely contribute to the continued burden of Vaccine Preventable Diseases (VPDs) in India [29]. An estimated 1.2 million Indian children under the age of five years die every year, the highest rate in the world [30]. Registering births will help track children to ensure they receive essential vaccinations.

The positive correlation between institutional deliveries and birth registration presents a clear pathway towards achieving universal birth registration and targeted health & social welfare policies in India. By focusing on targeted interventions in high-burden states, addressing social barriers, strengthening health care systems, and leveraging existing schemes like Janani Suraksha Yojana (JSY), India can unlock the potential of institutional deliveries to ensure that every child has their birth registered, thus paving the way for a brighter future. This necessitates a collaborative effort involving government agencies, health care providers, civil society organizations, and communities, all working together to bridge the gap and secure this fundamental right for all children.

## Data Availability

We hereby confirm that all authors have access to the data on which this publication is based.

## Future Scope of Research

The present study provides valuable insights into the relationship between institutionalization of births and birth registration in India. The study also provides a foundation for studying the interplay between wealth index and institutional deliveries. Future research could delve more deeper into sub-group analysis incorporating categories of place of delivery: institutional (public, private, or others) and non-institutional. Further, the impact of the type of health insurance availed by the households on institutional deliveries may be explored in future research.

## Limitations of the Study

The present study relied on secondary data from the NFHS-4 and the NFHS-5. While the sample size of the study is large, the data is self-reported and may be prone to recall bias.

## References

[r1] Office of Registrar General & Census Commissioner I (ORGI). The Registration of Births and Deaths Act. 1969. 1969. Office of Registrar General & Census Commissioner.

[r2] United Nations. Sustainable Development Goals. United Nations. 2015.

[r3] Selim L. What Is Birth Registration and Why Does It Matter? 2019. United Nations Children’s Fund (UNICEF).

[r4] Horton R, Lo S. Planetary health: A new science for exceptional action. Lancet. 2015;386(10007):1921–1922. doi:10.1016/S0140-6736(15)61038-8. 26188746

[r5] Setel PW, Macfarlane SB, Szreter S, et al. A scandal of invisibility: Making everyone count by counting everyone. Lancet. 2007;370(9598):1569–1577. doi:10.1016/S0140-6736(07)61307-5. 17992727

[r6] Bhatia A, Donger E, Bhabha J. Without an aadhaar card nothing could be done: A mixed methods study of biometric identification and birth registration for children in Varanasi, India. Inf Technol Dev. 2021;27(1):129–149. 10.1080/02681102.2020.1840325.

[r7] Kumari N. An assessment of birth registration system and factors affecting in India and its states. Online J Public Health Inform. 2019;11(1):e332. doi:10.5210/ojphi.v11i1.9813.

[r8] Mohanty I, Gebremedhin TA. Maternal autonomy and birth registration in India: Who gets counted? PLoS One. 2018;13(3):e0194095. doi:10.1371/journal.pone.0194095. 29534081 PMC5849310

[r9] Kumar K, Saikia N. Determinants of birth registration in India: Evidence from NFHS 2015–16. PLoS One. September 9, 2021;16:e0257014. doi:10.1371/journal.pone.0257014. 34473807 PMC8412296

[r10] Bhatia A, Kim R, Subramanian SV. Birth registration in India: Are wealth inequities decreasing? SSM Popul Health. 2021;13:100728. doi:10.1016/j.ssmph.2021.100728. 33532538 PMC7823051

[r11] Bhusal UP. Predictors of wealth-related inequality in institutional delivery: A decomposition analysis using Nepal multiple indicator cluster survey (MICS) 2019. BMC Public Health. 2021;21(1):2246. doi:10.1186/s12889-021-12287-2. 34893047 PMC8665495

[r12] Zere E, Oluwole D, Kirigia JM, Mwikisa CN, Mbeeli T. Inequities in skilled attendance at birth in Namibia: A decomposition analysis. BMC Pregnancy Childbirth. 2011;11(1):34. doi:10.1186/1471-2393-11-34. 21569585 PMC3118957

[r13] Saha R, Paul P. Institutional deliveries in India’s nine low performing states: Levels, determinants and accessibility. Glob Health Action. 2021;14(1):2001145. doi:10.1080/16549716.2021.2001145. 34914883 PMC8682830

[r14] Kumar R, Mandava S. Institutional deliveries in India: A study of associates and inequality. Int J Soc Econ. 2022;49(5):726–743. doi:10.1108/IJSE-08-2021-0444.

[r15] Paleker M, Boggs D, Jackson D, Day LT, Lawn JE. Closing the birth registration gap for *every newborn* facility birth: Literature review and qualitative research. Glob Health Action. 2023;16(1):2286073. doi:10.1080/16549716.2023.2286073. 38085000 PMC10795615

[r16] Jackson D, Wenz K, Muniz M, et al. Civil registration and vital statistics in health systems. Bull World Health Organ. 2018;96(12):861–863. doi:10.2471/BLT.18.213090. 30505035 PMC6249696

[r17] International Institute of Population Sciences (IIPS). National Family Health Survey (NFHS-4), 2015–16: India. 2017. International Institute of Population Sciences (IIPS).

[r18] International Institute of Population Sciences (IIPS). National Family Health Survey. 2021:*2019–21*. NFHS-5. International Institute of Population Sciences (IIPS).

[r19] The World Bank. National Family Health Survey (NFHS-4) 2015-16—IPUMS Subset. The World Bank.

[r20] StataCorp. Stata statistical software: Release 18. Published 2023.

[r21] Lorenz M, Aisch G, Kokkelink D. Datawrapper: Create charts and maps [Software]. Published 2012.

[r22] Ministry of Health and Family Welfare (MOHFW). Janani Suraksha Yojana. (JSY). Ministry of Health and Family Welfare (MOHFW). 2005.

[r23] Jeong J, Bhatia A, Fink G. Associations between birth registration and early child growth and development: Evidence from 31 low- and middle-income countries. BMC Public Health. 2018;18(1):673. doi:10.1186/s12889-018-5598-z. 29848302 PMC5977554

[r24] Wendt A, Hellwig F, Saad GE, et al. Birth registration coverage according to the sex of the head of household: An analysis of national surveys from 93 low- and middle-income countries. BMC Public Health. 2022;22(1):1942. doi:10.1186/s12889-022-14325-z. 36261798 PMC9583473

[r25] Dhirar N, Dudeja S, Khandekar J, Bachani D. Childhood morbidity and mortality in India—analysis of National Family Health Survey 4 (NFHS-4) findings. Indian Pediatr. 2018;55(4):335–338. 10.1007/s13312-018-1276-6. 29726828

[r26] Ministry of Health and Family Welfare Govt. of India. Guidelines for Janani Shishu Suraksha Karyakram. 2011. Ministry of Health and Family Welfare (MOHFW).

[r27] Muzzi M. UNICEF Good Practices in Integrating Birth Registration into Health Systems (2000–2009); Case Studies: Bangladesh, Brazil, the Gambia and Delhi, India. 2010. United Nations Children’s Fund (UNICEF).

[r28] UNICEF. Immunization and Child Health. One of the Most Effective and Cost-Effective Ways to Protect Children’s Lives and Futures. 2019. United Nations Children’s Fund (UNICEF).

[r29] Schueller E, Nandi A, Summan A, et al. Public finance of universal routine childhood immunization in India: District-level cost estimates. Health Policy Plan. 2022;37(2):200–208. doi:10.1093/heapol/czab114. 34522955 PMC8826633

[r30] Liu L, Villavicencio F, Yeung D, et al. National, regional, and global causes of mortality in 5–19-year-olds from 2000 to 2019: A systematic analysis. Lancet Glob Health. 2022;10(3):e337–e347. doi:10.1016/S2214-109X(21)00566-0. 35180417 PMC8864304

